# Regional gray matter alterations in anorexia nervosa: implications for subtype heterogeneity and clinical severity

**DOI:** 10.1038/s41380-026-03757-3

**Published:** 2026-07-15

**Authors:** Haidar Alzaid, Martin Bendszus, Hans-Christoph Friederich, Joe J. Simon

**Affiliations:** 1 Department of Neuroradiology, Heidelberg University Hospital, Heidelberg, Germany; 2 Department of General Internal Medicine and Psychosomatics, Heidelberg University Hospital, Heidelberg, Germany

**Keywords:** Neuroscience, Psychiatric disorders

## Abstract

Evidence indicates a global reduction in gray matter volume (GMV) in anorexia nervosa (AN) due to malnutrition. However, the specific regional GMV alterations that could contribute to the distinct subtypes and clinical presentations of AN remain poorly understood. This study aims to identify regional GMV alterations in AN and its subtypes, and investigate their association with clinical measures in a large sample. The study included 78 adult females with AN (57 restrictive AN-R and 21 binge/purge AN-BP subtype) and 94 healthy females. Voxel-based morphometry was used to compare regional GMV between groups, including AN subtypes. Exploratory correlation of regional GMV with BMI, age of illness onset, illness duration and EDE-Q was performed within the AN group. All analyses were adjusted for total GMV and age. Patients with AN displayed a mixed pattern of higher and lower regional GMV, including limbic, visual and executive regions, with distinct patterns observed in each subtype. Moreover, higher regional GMV was observed in AN-BP compared to AN-R in the right thalamus as well as temporal and occipital regions. Importantly, global volumetric measures did not differ between AN subtypes, indicating that these regional effects primarily reflect localized rather than global changes. In the entire AN group, exploratory analyses revealed that regional GMV correlated negatively with BMI in limbic structures as well as with EDE-Q eating concern subscore in the right insula. Taken together, diverse GMV alterations in acute AN likely reflect neurobiological differences underlying subtype heterogeneity, providing insight into their distinct clinical presentations and potential trait markers.

## Introduction

Anorexia nervosa (AN) is an eating disorder mainly characterized by persistent behavior to restrict energy intake, an intense fear of gaining weight and a distorted body image [1]. AN has a poor long-term prognosis [2, 3], high mortality rate [4] and is associated with numerous medical complications and psychological comorbidities [5]. While current evidence-based treatments can be effective, particularly with early intervention, treatment of AN remains challenging, with a significant risk of chronicity and relapse [6]. Although the pathogenesis of AN remains poorly understood, previous studies found alterations in brain circuits related to the development as well as maintenance of AN [7–10].

Structural neuroimaging studies have consistently shown a widespread brain volume reduction in patients with acute AN [11–13], which normalizes, at least partially, following short- [11] or long-term weight restoration [12]. This widespread volume loss presents a significant challenge in analyzing brain morphometry in AN, as it can potentially mask subtle, localized structural differences that might serve as trait markers for the disorder. To address this issue, it is essential to adjust for global brain volume reduction before making inferences about morphological trait markers. This adjustment typically involves including GMV as a covariate of no interest in statistical analyses [14]. Using this approach, several studies were able to reveal regional GMV alterations in AN [15–19]. However, many of these studies were constrained by small sample sizes, resulting in inconsistent findings across previous research. The largest voxel-based morphometry (VBM) study to date revealed a widespread, mostly symmetric pattern of regional GMV reduction in patients with AN, involving several cortical regions, the hippocampi, thalami and cerebellum [19]. Other commonly reported findings include GMV reduction in the cingulate gyrus and associated mesolimbic structures [16–18, 20]. Additionally, a study reported higher GMV in the left orbitofrontal cortex and the right anterior insula in patients with acute AN, as well as in recovered patients with AN, suggesting trait-related alterations [21]. However, other studies investigating weight-recovered patients with AN or patients with atypical AN characterized by higher body weight failed to find morphological alterations, arguing against the presence of structural trait markers [11, 12, 22].

AN is classified into two subtypes based on the primary methods of weight control: the restrictive subtype (AN-R) and the binge-eating/purging subtype (AN-BP). Whereas individuals with AN-R manage their weight through severe calorie restriction, those with AN-BP engage in binge-eating episodes followed by purging behaviors, such as self-induced vomiting, or the misuse of laxatives, diuretics and enemas [23]. Furthermore, AN-BP is associated with more severe eating-disorder pathology [24, 25], along with a higher prevalence of comorbid mental disorders, suicidality and impulsivity [26–28]. As a result, AN-BP is generally linked to a longer illness duration and worse clinical outcome compared to AN-R [29, 30]. Despite these critical differences, research on morphological variations between AN subtypes remains limited. The rise of large-scale multi-site studies has advanced the investigation of morphological abnormalities associated with AN by enhancing statistical power, enabling more robust and generalizable findings. However, while a general pattern of reduced GMV has been observed across both subtypes of AN, it remains unclear whether meaningful differences exist between the two. Although event-related fMRI studies have revealed differences in neural processing related to reward mechanisms, body image perception, and executive functioning between AN subtypes [31–33], research on morphological differences between the subtypes remains scarce. Additionally, the frequently observed diagnostic crossover between subtypes [34, 35] further complicates efforts to identify distinct morphological features. A multi-site study by Tose and colleagues [19] compared 58 patients with AN-R with 45 patients with AN-BP but failed to find significant differences in GMV between groups. This is in line with three previous studies that also found no significant GMV differences between subtypes in unmedicated and recovered patients with AN, though these studies involved smaller samples [36–38]. In the largest multi-site study to date, Walton and colleagues [13] employed volume- and surface-based metrics in a sample of 685 patients with AN. Although they found no direct effects of subtype on the observed results, sites with a higher proportion of patients with AN-R exhibited reduced insula thickness as well as reduced volume in the putamen and nucleus accumbens. This is somewhat consistent with a recent study by Yu and colleagues [39], who found that in a sample of 65 patients with AN, differences in the frontal lobe volumes between patients and healthy controls were driven by those with AN-R, while lower subcortical volumes were associated with the AN-BP group. However, this study did not directly compare the two subtypes. Taken together, current findings on GMV alterations associated with AN subtype are inconclusive, emphasizing the need for further research into their neurobiological differences.

To address inconsistencies observed in previous VBM studies, this study investigated regional GMV alterations in a large cohort of patients with AN, while accounting for global brain volume reduction. We examined differences between AN subtypes to elucidate their neurobiological characteristics. This approach also aimed to identify morphometric alterations across AN that could serve as trait markers for the disorder. Furthermore, we conducted an exploratory correlation analysis to examine the association between regional GMV alterations and eating disorder psychopathology as well as symptom severity, enhancing our understanding of how clinical presentation relates to brain structure in this population. This study is the first large-scale investigation of morphological differences between AN subtypes, using data from a single site, which may provide critical insights into their structural brain characteristics. The findings are expected to advance our understanding of how subtype-specific brain alterations contribute to the disorder and could inform more personalized diagnostic and therapeutic strategies for AN.

## Methods

### Participants

A total of 172 German adult women were included in the study, including 78 with acute AN (BMI  <  17.5 kg/m^2^, mean age = 23 years) and 94 healthy normal-weight controls (18.5  ≤  BMI  <  25 kg/m^2^, mean age = 24 years). The AN group included 57 individuals with the restrictive subtype and 21 with the binge/purge subtype. Patients with AN were recruited from the Department of General Internal Medicine and Psychosomatics at the University Hospital of Heidelberg. Patients had to meet the diagnostic criteria for AN (Diagnostic and Statistical Manual of Mental Disorders IV criteria) and have a BMI between 13 and 17.5 kg/m^2^. All diagnoses were established with the Structured Clinical Interview for DSM IV by trained clinical psychotherapists [40]. Screening for medical and psychiatric comorbidities followed the SCID procedures, and diagnostic decisions considered the interview, clinical notes, and patient history. Normal-weight controls had a BMI between 18.5 and 25 kg/m^2^ and no lifetime or current medical conditions that could potentially affect appetite or weight. Exclusion criteria for all participants were a history of head injury or surgery, neurological disorder, psychosis, bipolar disorder, current or lifetime substance abuse, borderline personality disorder, or the current use of psychotropic medications, except for antidepressants (s. supplementary table S1). Comorbidities were allowed in the AN group. Major depressive disorder was not listed as an exclusion criterion, given its high lifetime prevalence in AN (50–75%, [1, 41]) and in the general female population, which would have precluded a representative AN sample and yielded an over-selected control group. However, none of the included controls met SCID criteria for current major depressive disorder. Nineteen patients met criteria for depression, two for panic disorder, and one for obsessive compulsive disorder. Subgroup counts and further details are provided in Supplementary Table S2. Eating disorder symptoms were assessed in all participants using the Eating Disorder Examination Questionnaire EDE-Q [42].

### Image acquisition

Structural T1-weighted images MPRAGE were acquired with a 3 T scanner (Siemens Trio, Erlangen, Germany). The images were included from 3 fMRI studies and included two acquisition protocols: Protocol 1: TR  =  1570 ms, TE  =  2.63 ms; Protocol 2: TR  =  1900 ms, TE  =  2.52 ms. The remaining parameters were identical across protocols: flip angle = 7°; voxel size: 1 mm × 1 mm × 1 mm, matrix = 256 × 256, 192 slices with a slice thickness = 1 mm and no gap. The distribution of acquisition protocols across groups is provided in Supplementary Table S3.

### Voxel-based morphometry

The analysis was performed using the CAT12 toolbox implemented in Statistical Parametric Mapping (SPM) 12 [43–45]. Images were reoriented and converted to the Nifti format. Subsequently, images were visually inspected to assess the quality of image acquisition. The preprocessing pipeline included bias field correction, affine-registration and skull stripping. Images were then segmented into grey matter (GM), white matter (WM) and cerebrospinal fluid (CSF) maps. After segmentation, each individual GM map was normalized to the standard Montreal Neurological Institute (MNI) space using Diffeomorphic Anatomical Registration through Exponentiated Lie Algebra (DARTEL) method. The normalized GM and WM components were modulated by multiplying by the non-linear part of the deformation field at the DARTEL step, to generate the relative GM and WM volumes (WMV). The GMV/WMV images were spatially smoothed with an 8 mm FWHM isotropic Gaussian kernel. Global volumetric measures, including total intracranial volume (TIV), total brain volume (TBV), GMV, WMV and CSF, were calculated and compared between groups. Total GMV was used as nuisance covariate in each VBM analysis to adjust for global effects. We applied threshold-free cluster enhancement (TFCE) to the VBM results using 5000 permutations. This method combines focal and broad effects, improving the sensitivity of the analysis [46]. Clusters with an extent threshold > 10 voxels and a family wise error corrected (FWE) *p* value < 0.05 were considered significant. Localization of significant findings was done using the Automated Anatomical Labelling atlas 3 (AAL3) and verified through visual inspection.

### Statistical analysis

Statistical analysis of the clinical and extracted volumetric measures was performed using R (version 4.1.3). Normality of continuous variables was assessed using the Shapiro–Wilk test prior to parametric analyses. All variables met the assumptions for parametric testing. Clinical measures were compared between HC and patients with AN and between AN subtypes using two-sample t-tests. Global volumetric measures were compared between groups using ANCOVA (analysis of covariance) to adjust for covariates of no interest. In the comparison between HC and patients with AN, TIV and age were included as covariates of no interest, with TIV specifically included to account for variations in head size. When comparing AN subtypes, we included TIV, age, BMI, illness duration, and age of illness onset as covariates of no interest. To account for multiple testing across five global volumetric parameters and two group comparisons (total of 10 tests), a Bonferroni correction was applied, with the significance threshold adjusted accordingly.

Group comparisons of regional differences in GMV were performed using two-sample t-tests. When comparing HC with all patients with AN and each AN subtype, we included total GMV and age as nuisance covariates. When comparing AN subtypes directly, we included BMI, illness duration, and age of illness onset as additional covariates of no interest. Furthermore, a whole-brain regression analysis was performed within the entire AN-group to assess the relation between regional GMV and BMI, illness duration, age of illness onset as well as eating-disorder symptoms (EDE-Q mean score and subscales scores). In each regression analysis with BMI, illness duration, and age of illness, the other two parameters were included as covariates of no interest, e.g. correlation with BMI included illness duration and age of illness as covariates of no interest. As an additional exploratory analysis, we conducted the same regression analyses within each AN subtype separately. Since a substantial proportion of patients with AN were taking antidepressants at the time of examination, antidepressant intake was included as a covariate of no interest in all analyses. The acquisition protocol was likewise included as a covariate of no interest to account for potential methodological variability. Multiple comparisons were corrected separately within each individual neuroimaging analysis, while no correction was applied across different analyses at the overall study level.

## Results

### Demographic and clinical features

BMI was significantly lower in AN compared to HC (*p* < 0.001). EDE-Q mean score and subscores were significantly higher in AN compared to HC (*p* < 0.001). AN-BP had a significantly lower age of illness onset (*p* < 0.001) and longer illness duration (*p* < 0.05) compared to AN-R. The other parameters did not differ significantly between AN subtypes. The distribution of comorbidities did not differ between AN-R and AN-BP (see Supplementary Table S2). There was no difference in age between groups. The results are summarized in Table 1. The distribution of acquisition protocols did not differ between AN and HC. However, a significant difference in protocol distribution was observed between AN-R and AN-BP (see Supplementary Table S3).

**Table 1:** Clinical features.

Parameter	HC (*n* = 94)	AN (*n* = 78)	AN-R (*n* = 57)	AN-BP (*n* = 21)	HC vs. AN (*p* value)	AN-R vs. AN-BP (*p* value)
Age	23.5 ± 2.9	23.1 ± 4.9	22.7 ± 4.4	24.1 ± 5.8	ns	ns
BMI	21.4 ± 1.4	15.1 ± 1.4	15.0 ± 1.3	15.3 ± 1.6	<0.001	ns
Age of illness onset	–	18.1 ± 4.2	18.9 ± 4.3	15.9 ± 2.6	–	< 0.001
Illness duration (months)	–	64.8 ± 63.4	53.0 ± 57.1	97.1 ± 69.7	–	< 0.05
EDE-Q (total)	0.4 ± 0.5	3.2 ± 1.6	3.1 ± 1.6	3.4 ± 1.7	<0.001	ns
EDE-Q (restraint)	0.4 ± 0.7	3.2 ± 2.0	3.2 ± 2.0	3.1 ± 1.9	<0.001	ns
EDE-Q (eating concern)	0.1 ± 0.3	2.7 ± 1.7	2.7 ± 1.7	2.8 ± 1.7	<0.001	ns
EDE-Q (weight concern)	0.4 ± 0.9	3.1 ± 1.5	3.1 ± 1.4	3.2 ± 1.9	<0.001	ns
EDE-Q (shape concern)	0.6 ± 0.9	3.7 ± 1.7	3.7 ± 1.6	3.9 ± 1.8	<0.001	ns

All values are displayed as the mean ± standard deviation.

*HC* healthy controls, *AN* anorexia nervosa, *AN-R* anorexia nervosa – restrictive subtype, *AN-BP* anorexia nervosa - binge/purge subtype, *BMI* body mass index, *EDE-Q* Eating Disorder Examination Questionnaire, *ns* not significant.

Note: EDE-Q data were only available for 73/78 patients.

### Analysis of global volumetric measures

TBV, GMV and WMV were significantly lower and CSF was significantly higher in AN compared to HC (*p* < 0.001). There were no significant differences in global volumetric measures between AN subtypes. There was no significant difference in TIV between groups. Within the AN group, total GMV was positively correlated with BMI (R = 0.57; *p* < 0.001). There was no significant correlation between total GMV and the other parameters. The results are summarized in Table 2.

**Table 2:** Group comparison of global volumetric measures.

Volumetric parameter	HC	AN	AN-R	AN-BP	HC vs. AN (*p* value)	AN-R vs. AN-BP (*p* value)
GMV	686.3 ± 3.5	637.2 ± 3.9	627.0 ± 4.5	638.1 ± 8.0	< 0.001	ns
WMV	493.7 ± 2.6	475.2 ± 2.9	466.5 ± 3.4	471.8 ± 6.0	< 0.001	ns
CSF	240.3 ± 4.7	307.9 ± 5.1	306.4 ± 6.2	290.1 ± 11.0	< 0.001	ns
TBV	1179.9 ± 4.7	1112.4 ± 5.1	1093.5 ± 6.2	1109.8 ± 11.0	< 0.001	ns
TIV	1437.1 ± 10.7	1399.9 ± 11.7	1390.8 ± 15.4	1424.7 ± 25.4	ns	ns

All values are displayed as the mean (cm^3^) ±  standard error of the mean.

*HC* healthy controls, *AN* anorexia nervosa, *AN-R* anorexia nervosa – restrictive subtype, *AN-BP* anorexia nervosa - binge/purge subtype, *GMV* gray matter volume, *WMV* white matter volume, *CSF* cerebrospinal fluid volume, *TBV* total brain volume, *TIV* total intracranial volume, *ns* not significant.

### Voxel-based morphometry: Group comparisons

**Fig. 1:**
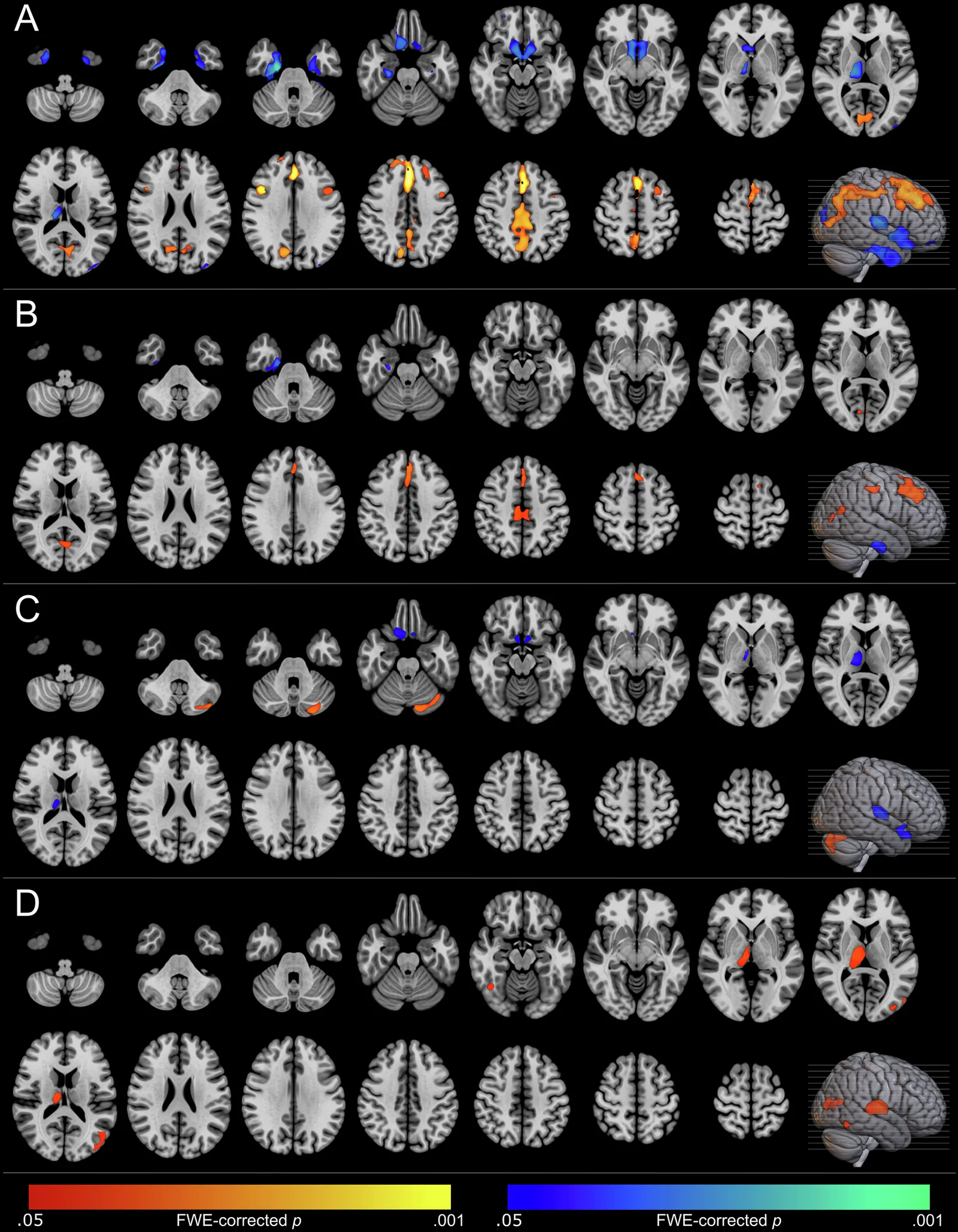
Results of the group comparisons displayed on the 1 mm MNI-152 template. All results are corrected for multiple comparisons using an FWE-corrected *p* value of < 0.05 (corrected within each analysis). **A:** AN entire cohort vs HC: Warm colors represent lower regional GMV and cool colors represent higher regional GMV in AN (adjusted for age, medication status, acquisition protocol and total GMV). **B:** AN-R vs HC: Warm colors represent lower regional GMV and cool colors represent higher regional GMV in AN-R (adjusted for age, medication status, acquisition protocol and total GMV). **C:** AN-BP vs HC: Warm colors represent lower regional GMV and cool colors represent higher regional GMV in AN-BP (adjusted for age, medication status, acquisition protocol and total GMV). **D:** AN-R vs AN-BP: Warm colors represent lower regional GMV in AN-R (adjusted for age, medication status, acquisition protocol, total GMV, BMI, age of illness onset and illness duration).

Our VBM analysis revealed a mixed pattern of GMV increases and decreases in AN compared to HC. Specifically, we observed decreased GMV in Patients with AN in prefrontal and cingulate regions, but increased GMV in patients with AN in limbic, temporal and occipital regions. When comparing HC with each subtype separately, some of these changes were observed in AN-R, while others were found in AN-BP, highlighting distinct neuroanatomical patterns in each subtype. Specifically, AN-R displayed a GMV reduction in prefrontal and cingulate regions as well as GMV enlargement in the parahippocampal and fusiform gyri, while AN-BP displayed GMV reduction in the cerebellum as well as GMV enlargement in the right thalamus as well as limbic and occipital regions. Direct comparison between AN subtypes revealed lower GMV in AN-R compared to AN-BP in the right thalamus and in several bilateral temporal and occipital regions. Detailed results of these group comparisons are provided in Table 3 and Fig. 1.

**Table 3:** Results of VBM group comparisons.

Cluster	Peak region	MNI coordinates	*p* value (FWE-corrected)	Cluster size (voxels)	Cluster regions
**HC** > **AN**					
1	Superior frontal gyrus (medial, L)	−2 × 34 × 38	0.002	11429	Superior frontal gyrus (medial, L R); supplementary motor area (L R); middle cingulate gyrus (L R); paracentral lobule (L R); anterior cingulate cortex (supracallosal, L); precuneus (L R); cuneus (L R); calcarine fissure (L R).
	Superior frontal gyrus (medial, L)	−2 × 24 × 42	0.002	Subpeak	
	Supplementary motor area (L)	−2 × 14 × 50	0.003	Subpeak	
	Supplementary motor area (L)	0 × 12 × 44	0.004	Subpeak	
2	Inferior frontal gyrus (opercular part R)	44 ×14 × 32	0.004	518	Inferior frontal gyrus (opercular and triangular part, R); middle frontal gyrus (R); precentral gyrus (R).
3	Superior frontal gyrus (dorsolateral, L)	−24 × 36 × 39	0.034	283	Superior frontal gyrus (dorsolatera, L); middle frontal gyrus (L).
	Middle frontal gyrus (L)	−26 × 32 × 38	0.034	Subpeak	
**HC** < **AN**					
1	Parahippocampal gyrus (R)	21 × −16 × −33	0.002	2377	Parahippocampal gyrus (R); fusiform gyrus (R); hippocampus (R); amygdala (R); inferior temporal gyrus (R).
	Parahippocampal gyrus (R)	22 × −12 × −32	0.002	Subpeak	
	Parahippocampal gyrus (R)	22 × −22 × −30	0.002	Subpeak	
	Fusiform gyrus (R)	32 × −15 × −39	0.006	Subpeak	
2	Nucleus accumbens (R)	6 × −2 × −8	0.006	4062	Nucleus accumbens (L R); caudate nucleus (L R); olfactory cortex (L R); gyrus rectus (L R); medial orbital gyrus (L R); posterior orbital gyrus (L).
	Nucleus accumbens (R)	8 × 10 × −14	0.006	Subpeak	
	Nucleus accumbens (R)	9 × 18 × −10	0.006	Subpeak	
	Nucleus accumbens (L)	−4 × −4 × −14	0.009	Subpeak	
3	Thalamus (R)	16 × −16 × 6	0.006	1308	Thalamus (R).
	Thalamus (R)	9 × −10 × 0	0.007	Subpeak	
	Thalamus (R)	6 × −6 × −2	0.008	Subpeak	
	Thalamus (R)	15 × −21 × 15	0.008	Subpeak	
4	Fusiform gyrus (L)	−26 × −9 × −45	0.017	1374	Fusiform gyrus (L); parahippocampal gyrus (L); hippocampus (L); amygdala (L) inferior temporal gyrus (L).
	Fusiform gyrus (L)	−28 × −15 × −39	0.018	Subpeak	
	Fusiform gyrus (L)	−32 × −18 × −38	0.018	Subpeak	
	Parahippocampal gyrus (L)	−26 × −10 × −33	0.018	Subpeak	
5	Middle occipital gyrus (L)	−40 × −92 × 12	0.023	331	Middle occipital gyrus (L).
	Middle occipital gyrus (L)	−33 × −93 × 22	0.025	Subpeak	
	Middle occipital gyrus (L)	−36 × −90 × 24	0.026	Subpeak	
	Middle occipital gyrus (L)	−45 × −87 × 16	0.027	Subpeak	
**HC** > **AN-R**					
1	Superior frontal gyrus (medial, L)	−2 × 34 × 39	0.021	1451	Superior frontal gyrus (medial, L R); supplementary motor area (L R); middle cingulate gyrus (L R); anterior cingulate cortex (supracallosal, L).
	Middle cingulate gyrus (L)	0 × 22 × 39	0.023	Subpeak	
	Supplementary motor area (L)	−2 × 21 × 48	0.027	Subpeak	
	Supplementary motor area (L)	−2 × 22 × 52	0.027	Subpeak	
2	Middle cingulate gyrus (R)	8 × −22 × 45	0.027	657	Middle cingulate gyrus (L R); supplementary motor area (L R); paracentral lobule (L R).
	Paracentral lobule (L)	−6 × −24 × 45	0.033	Subpeak	
3	Calcarine fissure (L)	−3 × −68 × 16	0.034	548	Calcarine fissure (L R); precuneus (L R); cuneus (L R); lingual gyrus (L R).
	Calcarine fissure (R)	4 × −66 × 18	0.034	Subpeak	
	Calcarine fissure (R)	6 × −81 × 3	0.047	Subpeak	
	Calcarine fissure (R)	9 × −78 × 10	0.047	Subpeak	
**HC** < **AN-R**					
1	Parahippocampal gyrus (R)	24 × −16 × −28	0.007	736	Parahippocampal gyrus (R); fusiform gyrus (R); hippocampus (R).
	Fusiform gyrus (R)	34 × −24 × −33	0.031	Subpeak	
	Fusiform gyrus (R)	33 × −15 × −40	0.032	Subpeak	
	Fusiform gyrus (R)	28 × −12 × −42	0.033	Subpeak	
**HC** > **AN-BP**					
1	Crus II of cerebellar Hemisphere (L)	−28 × −84 × −34	0.023	1371	Crus I of cerebellar hemisphere (L). Crus II of cerebellar hemisphere (L).
	Crus II of cerebellar Hemisphere (L)	−33 × −82 × −36	0.023	Subpeak	
	Crus I of cerebellar Hemisphere (L)	−14 × −81 × −24	0.024	Subpeak	
	Crus I of cerebellar Hemisphere (L)	−40 × −78 × −39	0.024	Subpeak	
**HC** < **AN-BP**					
1	Thalamus (R)	8 × −6 × 0	0.023	921	Thalamus (R).
	Thalamus (R)	16 × −15 × 8	0.025	Subpeak	
	Thalamus (R)	15 × −22 × 16	0.031	Subpeak	
	Thalamus (R)	6 × −3 × −9	0.041	Subpeak	
2	Middle orbital gyrus (R)	15 × 21 × −26	0.027	841	Middle orbital gyrus (R); nucleus accumbens (L R); caudate nucleus (R); olfactory cortex (L R); gyrus rectus (L R); posterior orbital gyrus (R).
	Gyrus rectus (R)	9 × 20 × −27	0.028	Subpeak	
	Olfactory cortex (R)	4 × 9 × −21	0.036	Subpeak	
	Nucleus accumbens (R)	9 × 18 × −10	0.037	Subpeak	
3	Middle occipital gyrus (L)	−40 × −92 × 14	0.049	11	Middle occipital gyrus (L).
**AN-R** < **AN-BP**					
1	Thalamus (R)	14 × −16 × 4	0.034	1974	Thalamus (R)
	Thalamus (R)	14 × −18 × 9	0.034	Subpeak	
	Thalamus (R)	18 × −32 × 8	0.044	Subpeak	
2	Middle occipital gyrus (L)	−39 × −86 × 14	0.046	799	Middle occipital gyrus (L); middle temporal gyrus (L).
	Middle occipital gyrus (L)	−48 × −82 × 15	0.046	Subpeak	
	Middle occipital gyrus (L)	−52 × −78 × 8	0.047	Subpeak	
	Middle temporal gyrus (L)	−50 × −68 × 16	0.047	Subpeak	
3	Fusiform gyrus (R)	42 × −60 × −16	0.048	158	Fusiform gyrus (R); inferior temporal gyrus (R).
4	Inferior occipital gyrus (R)	27 × −87 × −9	0.049	33	Inferior occipital gyrus (R); lingual gyrus (R).

Each significant cluster is defined by the region with the peak *p* value, and up to 3 subpeaks are reported per cluster. All regions within the clusters are listed according to the AAL3 atlas and verified through visual inspection.

*HC*, healthy controls; *AN*, anorexia nervosa; *AN-R*, anorexia nervosa – restrictive subtype; *AN-BP*, anorexia nervosa - binge/purge subtype; *R*, right hemisphere; *L*, left hemisphere.

### Voxel-based morphometry: Exploratory regression analyses

**Fig. 2:**
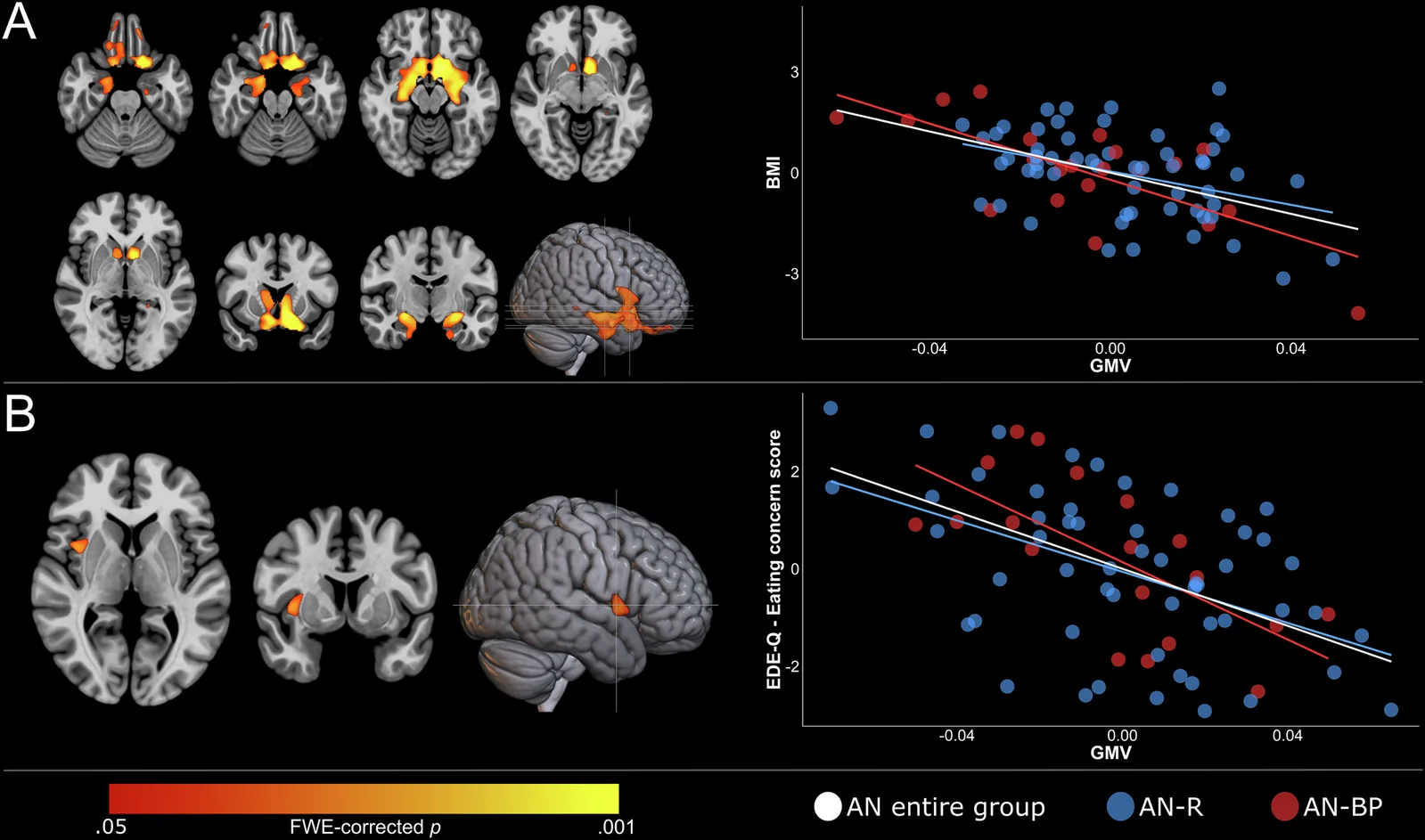
Left side: Results of the whole-brain regression analyses within the entire AN group, with all results corrected for multiple comparisons using an FWE-corrected *p*-value of < 0.05 (corrected within each analysis). Right side: The plotted values represent standardized residuals of GMV extracted from the entire significant cluster identified in the regression analysis after adjustment for covariates of no interest. **A:** Negative correlation between BMI and regional GMV, adjusted for age, illness duration, age of illness onset, medication status, acquisition protocol and total GMV. **B:** Negative correlation between EDE-Q eating concern score and regional GMV, adjusted for age, medication status, acquisition protocol and total GMV.

The exploratory regression analysis revealed a significant negative relation between GMV and BMI in several limbic structures in AN. There was also a significant negative relation between GMV and EDE-Q eating concern subscore in the right insula. We found no significant correlation with age of illness onset, illness duration, the mean EDE-Q score or the other EDE-Q subscores. Detailed results of the regression analysis can be found in Table 4 and Fig. 2. The exploratory regression within AN subtypes revealed a significant negative correlation between BMI and GMV in the right orbitofrontal regions in AN-BP (see supplementary material), but no significant correlation in the AN-R group. The control analysis between medicated and unmedicated patients with AN revealed no significant differences.

**Table 4:** Results of VBM exploratory regression analyses.

Cluster	Peak region	MNI coordinates (X Y Z)	*p* value (FWE-corrected)	Cluster size (voxels)	Cluster regions
**Negative correlation with BMI in AN**					
1	Nucleus accumbens (L)	−10 × 10 × −14	0.003	9617	Nucleus accumbens (L R); caudate nucleus (L R); olfactory cortex (L R); gyrus rectus (L R); medial orbital gyrus (L R); posterior orbital gyrus (L); insula (L): amygdala (L R); hippocampus (L R); parahippocampal gyrus (L R).
	Olfactory cortex (L)	−8 × 4 × −18	0.003	Subpeak	
	Medial orbital gyrus (L)	−18 × 15 × −21	0.004	Subpeak	
	Amygdala (L)	−14 × −2 × −14	0.005	Subpeak	
**Negative correlation with EDE-Q eating concern score in AN**					
1	Insula (R)	40 × 8 × 2	0.013	360	Insula (R); inferior frontal gyrus (opercular part R); rolandic operculum (R).

Each significant cluster is defined by the region with the peak *p* value, and up to 3 subpeaks are reported per cluster. All regions within the clusters are listed according to the AAL3 atlas and verified through visual inspection.

*HC*, healthy controls; *AN*, anorexia nervosa; *BMI*, body mass index; *EDE-Q*, Eating Disorder Examination Questionnaire; *R*, right hemisphere; *L*, left hemisphere.

## Discussion

In this study, we performed a comprehensive analysis of regional GMV alterations in a large sample of patients with acute AN, including comparisons between restrictive and binge/purge subtypes. Our findings revealed an intriguing pattern of GMV reductions and enlargements in patients with AN, with distinct patterns observed in each subtype. Furthermore, we found that higher GMV in the mesolimbic reward system was associated with lower body weight, whereas lower GMV in the right insula was linked to greater severity of eating disorder psychopathology. Since direct comparisons of GMV between AN subtypes are scarce, these results provide important insights into the neurobiological differences underlying the subtypes, which may inform targeted therapeutic approaches and future research into the mechanisms of AN.

When comparing patients with healthy controls, we observed GMV reductions in key regions such as the supplementary motor cortex (SMA) and midcingulate cortex (MCC) [19, 47], reinforcing earlier findings of abnormal activity in these areas in AN [48, 49]. In fact, a recent meta-analysis highlighted structural and functional alterations in the MCC as one of the most robust and reproducible neuroimaging findings across studies of AN [20]. Both the SMA and MCC are integral to cognitive flexibility [50], a cognitive domain that is frequently impaired in AN [51]. Thus, the morphological changes observed in these regions may contribute to a core feature of AN, cognitive-behavioral inflexibility [51]. Reductions of GMV in AN were also observed in other areas of the frontal cortex, including the right inferior frontal gyrus and bilateral superior frontal gyrus. These findings align with previous studies reporting reduced GMV [16, 19] and altered functional connectivity [52] in these regions, which are thought to underlie deficits in cognitive control in AN [52]. Furthermore, patients with AN also displayed reduced GMV in regions involved in visual processing. This is in line with previous reports of lower GMV in regions such as the bilateral fusiform and parahippocampal gyri [53]. Both regions are involved in high-level visual processing and visual categorization, including recognition of faces and bodies [54, 55]. Accordingly, morphological alterations of these regions could contribute to body image distortion in AN. Supporting this, an fMRI-study revealed reduced activation in these regions in patients with AN when viewing their own body, but increased activation when viewing another woman’s body [56]. Furthermore, increased activation in the fusiform gyrus in response to visual food stimuli during a state of hunger has also been observed in AN [57, 58]. Taken together, the structural alterations observed in patients with AN compared to healthy controls may point towards a neurobiological basis for the commonly observed impairments in cognitive flexibility and body image distortions in AN. Since we adjusted for global effects using total GMV, these results may reflect trait-specific alterations associated with the disorder.

Interestingly, we found that patients with AN display higher GMV in areas such as the nucleus accumbens and orbitofrontal cortex, regions central to the brain’s reward system [59, 60]. This is in line with the common observation of altered reward processing in AN [61] as well as with previous studies that found increased GMV in these regions in acutely ill and recovered patients with AN [15, 21, 53, 62]. This observation may reflect a pathophysiological trait marker of AN, as increased structural and functional connectivity within the reward network has been found in both acute AN as well as following weight restoration. This suggests that atypical connectivity in the fronto-accumbal network may persist despite weight normalization [63]. Similarly, higher structural connectivity between the insula, orbitofrontal cortex and ventral striatum has been reported in both in AN and bulimia nervosa [64]. Collectively, this evidence highlights the critical role of the brain’s reward system in the pathophysiology of AN. Moreover, interventions targeting this system, such as deep brain stimulation of the nucleus accumbens, have shown promise as effective treatments for severe and chronic cases of AN [65].

When comparing AN subtypes to HC, we found that patients with AN-R display GMV reductions in the superior frontal gyrus and cingulate gyrus, alongside GMV increases within the limbic system, specifically in the parahippocampal gyrus. This pattern aligns with the hypothesis of reduced top-down regulation, which is thought to drive weight-related anxiety and reinforce food restriction [61]. Supporting this, an fMRI study found increased activation in the amygdala and decreased activation in the prefrontal cortex in AN-R compared to both HC and AN-BP in response to images of overweight bodies [33]. On the other hand, we found that in patients with AN-BP, GMV alterations were primarily observed in regions associated with reward and visual processing as well as attention. This pattern resembles changes in the attentional network observed in bulimia nervosa, such as enlargement of occipital gyri and volume reduction in the precuneus [66], along with corresponding hyper- and hypoactivation, respectively [67]. Our findings align with commonly observed attentional deficits and impulsivity in AN-BP, possibly contributing to binge-eating episodes [68]. Interestingly, lower GMV was also observed in the left cerebellum (Crus I & II) in AN-BP compared to HC. The cerebellum is known to play a role in the pathophysiology of eating disorders with growing evidence of corresponding cerebellar abnormalities, including GMV reductions and functional alterations across multiple subregions in AN [19, 69]. This is consistent with the cerebellum´s role in modulating feeding behavior through direct and indirect connections with several key feeding control centers, such as the hypothalamus [70].

When directly comparing both subtypes, we found widespread reductions in GMV in AN-R compared to AN-BP. Although previous studies are inconsistent, reporting mixed results, including findings of no differences between subgroups [36–38], our results are in line with some previous studies that found reduced GMV in AN-R when compared to AN-BP. However, the specific regions observed differ, with observations of reduced thickness of regions within the brain reward system [13, 71], reduced GMV in prefrontal regions [39] or somatosensory regions [71] in AN-R when compared to AN-BP. These differences may be accounted for by methodological differences such as small sample sizes or particularly the use of TBV instead of total GMV to adjust for global effects. Taken together, the findings support the view that AN subtypes may not represent distinct entities, but might instead reflect variations along a continuum [72, 73]. However, given the relatively small subgroup samples, this interpretation should be viewed as preliminary and warrants confirmation in larger samples.

Our exploratory correlation analysis revealed a negative relation between BMI and regional GMV in a large cluster encompassing limbic and orbitofrontal Structures. This relation remained significant even after accounting for illness duration and age of illness onset. This is in line with previous observations of altered structural and effective connectivity between the ventral striatum and hypothalamus in AN [64], alongside structural alterations in the hypothalamus [74] that may contribute to altered reactivity to food stimuli [75, 76]. Moreover, adaptive alterations in the dopaminergic reward network are associated with chronic food restriction, resembling patterns observed in addiction disorders [77]. Considering the evidence of trait-related changes in the reward system, the structural alterations we observed may reflect adaptive responses to food restriction that further reinforce trait vulnerability. In contrast, the largest study on AN to date [13] reported a positive correlation between BMI and subcortical volume; however, as this analysis did not adjust for global brain volume reduction, the discrepancy with our findings is most likely attributable to global rather than region-specific effects. Furthermore, we found a negative relation between GMV in the right insula and eating-disorder related symptoms. This finding suggests that reduced GMV in the right insula may be associated with greater overall eating disorder psychopathology, which is supported by previous research highlighting the insula’s critical role in interoceptive awareness and energy balance regulation, functions that are often disrupted in individuals with eating disorders. For instance, alterations in insular function have been linked to impaired recognition of hunger and satiety cues, as well as with altered visceral interoception, potentially contributing to maladaptive eating behaviors [78, 79]. Finally, when analysing each AN subtype individually, we found a significant relation between GMV in orbitofrontal regions and BMI in AN-BP, but not in AN-R. This suggests that this relation might be driven by the binge-purge subtype. These regression findings are exploratory and should be interpreted with caution, requiring replication in independent samples.

Several limitations should be acknowledged. For example, the cross-sectional design of our study restricts our ability to draw causal inferences, particularly regarding whether the observed regional brain changes are adaptive effects of prolonged malnutrition or a predisposing factor. Future longitudinal studies are necessary to clarify the temporal relationship between these markers and the disorder, taking into account broader global changes over time. Furthermore, given that previous studies have shown that up to approximately 42% of patients with AN-R exhibit diagnostic crossover to binging and purging behaviors over time [35], longitudinal studies may provide valuable insights into the morphological changes associated with this transition. A further limitation stems from the multiple tests in our design, which introduces a residual risk of Type I error at the overall study level despite appropriate correction within each individual neuroimaging analysis using TFCE with whole-brain FWE correction (*p* < 0.05). This is compounded by the relatively small sample sizes within each AN subtype, particularly AN-BP, which may limit statistical power and the generalizability of our findings. As a result, the distinct patterns observed in each AN subtype likely reflect predominant rather than exclusive features. Further research with larger samples is necessary to confirm these observations and enhance our understanding of the neurobiological differences among AN subtypes. Another important limitation is that some patients were taking antidepressants, which can induce dynamic structural brain changes [80]. In addition, data were acquired using two slightly different acquisition protocols, which could introduce methodological variability. To mitigate these potential confounds, both medication status and acquisition protocol were included as covariates in all analyses, reducing the likelihood that the observed effects were driven by these factors.

In conclusion, our study identified a complex pattern of regional GMV alterations across limbic, visual, and executive brain regions, which varied by disease subtype, highlighting AN-related, trait-specific morphometric patterns. As global volumetric measures did not differ between subtypes, these findings suggest that the heterogeneity in AN is driven primarily by localized rather than global changes. Moreover, exploratory analyses suggested that GMV enlargement within the limbic reward system and the right insula was associated with lower body weight and more severe eating disorder psychopathology, respectively. This relationship appeared to be driven by the binge-purge subtype, indicating that structural brain alterations may contribute to the heterogeneity seen in AN subtypes and clinical presentations. This suggests a possible neurobiological contribution to the heterogeneity of the illness, highlighting that different therapeutic strategies might be warranted in the future.

## Supplementary information

Supplementary material

## Data Availability

The data used in this study are available from the corresponding author upon reasonable request.

## References

[1] American Psychiatric Association. Diagnostic and Statistical Manual of Mental Disorders (DSM-5®). Washington, DC: American Psychiatric Association; 2013.

[2] Herpertz-Dahlmann B, Dempfle A, Egberts KM, Kappel V, Konrad K, Vloet JA, et al. Outcome of childhood anorexia nervosa-The results of a five- to ten-year follow-up study. Int J Eat Disord. 2018;51:295–304. 10.1002/eat.22840 29451957

[3] Dobrescu SR, Dinkler L, Gillberg C, Rastam M, Gillberg C, Wentz E. Anorexia nervosa: 30-year outcome. Br J Psychiatry. 2020;216:97–104. 10.1192/bjp.2019.113 PMC7557598 31113504

[4] Smink FR, van Hoeken D, Hoek HW. Epidemiology, course, and outcome of eating disorders. Curr Opin Psychiatry. 2013;26:543–8. 10.1097/YCO.0b013e328365a24f 24060914

[5] Van Alsten SC, Duncan AE. Lifetime patterns of comorbidity in eating disorders: An approach using sequence analysis. Eur Eat Disord Rev. 2020;28:709–23. 10.1002/erv.2767 32748537

[6] Zipfel S, Giel KE, Bulik CM, Hay P, Schmidt U. Anorexia nervosa: aetiology, assessment, and treatment. Lancet Psychiatry. 2015;2:1099–111. 10.1016/S2215-0366(15)00356-9 26514083

[7] Simon JJ, Stopyra MA, Friederich HC. Neural processing of disorder-related stimuli in patients with anorexia nervosa: a narrative review of brain imaging studies. J Clin Med. 2019;8:1047. 10.3390/jcm8071047 PMC6678397 31323803

[8] Bulik CM, Coleman JRI, Hardaway JA, Breithaupt L, Watson HJ, Bryant CD, et al. Genetics and neurobiology of eating disorders. Nat Neurosci. 2022;25:543–54. 10.1038/s41593-022-01071-z PMC9744360 35524137

[9] Zhong S, Su T, Gong J, Huang L, Wang Y. Brain functional alterations in patients with anorexia nervosa: A meta-analysis of task-based functional MRI studies. Psychiatry Res. 2023;327:115358. 10.1016/j.psychres.2023.115358 37544086

[10] Phillipou A, Rossell SL, Castle DJ. The neurobiology of anorexia nervosa: a systematic review. Aust N Z J Psychiatry. 2014;48:128–52. 10.1177/0004867413509693 24194589

[11] Bernardoni F, King JA, Geisler D, Stein E, Jaite C, Nätsch D, et al. Weight restoration therapy rapidly reverses cortical thinning in anorexia nervosa: A longitudinal study. Neuroimage. 2016;130:214–22. 10.1016/j.neuroimage.2016.02.003 26876474

[12] King JA, Geisler D, Ritschel F, Boehm I, Seidel M, Roschinski B, et al. Global cortical thinning in acute anorexia nervosa normalizes following long-term weight restoration. Biol Psychiatry. 2015;77:624–32. 10.1016/j.biopsych.2014.09.005 25433902

[13] Walton E, Bernardoni F, Batury VL, Bahnsen K, Larivière S, Abbate-Daga G, et al. Brain structure in acutely underweight and partially weight-restored individuals with anorexia nervosa: a coordinated analysis by the ENIGMA Eating Disorders Working Group. Biol Psychiatry. 2022;92:730–8. 10.1016/j.biopsych.2022.04.022 PMC12145862 36031441

[14] Peelle JE, Cusack R, Henson RN. Adjusting for global effects in voxel-based morphometry: gray matter decline in normal aging. Neuroimage. 2012;60:1503–16. 10.1016/j.neuroimage.2011.12.086 PMC3898996 22261375

[15] Mishima R, Isobe M, Noda T, Tose K, Kawabata M, Noma S, et al. Structural brain changes in severe and enduring anorexia nervosa: A multimodal magnetic resonance imaging study of gray matter volume, cortical thickness, and white matter integrity. Psychiatry Res Neuroimaging. 2021;318:111393. 10.1016/j.pscychresns.2021.111393 34670165

[16] Friederich HC, Walther S, Bendszus M, Biller A, Thomann P, Zeigermann S, et al. Grey matter abnormalities within cortico-limbic-striatal circuits in acute and weight-restored anorexia nervosa patients. Neuroimage. 2012;59:1106–13. 10.1016/j.neuroimage.2011.09.042 21967727

[17] Mühlau M, Gaser C, Ilg R, Conrad B, Leibl C, Cebulla MH, et al. Gray matter decrease of the anterior cingulate cortex in anorexia nervosa. Am J Psychiatry. 2007;164:1850–7. 10.1176/appi.ajp.2007.06111861 18056240

[18] Gaudio S, Nocchi F, Franchin T, Genovese E, Cannatà V, Longo D, et al. Gray matter decrease distribution in the early stages of Anorexia Nervosa restrictive type in adolescents. Psychiatry Res. 2011;191:24–30. 10.1016/j.pscychresns.2010.06.007 21081268

[19] Tose K, Takamura T, Isobe M, Hirano Y, Sato Y, Kodama N, et al. Systematic reduction of gray matter volume in anorexia nervosa, but relative enlargement with clinical symptoms in the prefrontal and posterior insular cortices: a multicenter neuroimaging study. Mol Psychiatry. 2024;29:891–901. 10.1038/s41380-023-02378-4 PMC11176065 38246936

[20] Su T, Gong J, Tang G, Qiu S, Chen P, Chen G, et al. Structural and functional brain alterations in anorexia nervosa:A multimodal meta-analysis of neuroimaging studies. Hum Brain Mapp. 2021;42:5154–69. 10.1002/hbm.25602 PMC8449099 34296492

[21] Frank GK, Shott ME, Hagman JO, Mittal VA. Alterations in brain structures related to taste reward circuitry in ill and recovered anorexia nervosa and in bulimia nervosa. Am J Psychiatry. 2013;170:1152–60. 10.1176/appi.ajp.2013.12101294 PMC3789862 23680873

[22] Olivo G, Solstrand Dahlberg L, Wiemerslage L, Swenne I, Zhukovsky C, Salonen-Ros H, et al. Atypical anorexia nervosa is not related to brain structural changes in newly diagnosed adolescent patients. Int J Eat Disord. 2018;51:39–45. 10.1002/eat.22805 29215777

[23] American Psychiatric Association D, Association AP. *Diagnostic and statistical manual of mental disorders: DSM-5*, vol. 5. American psychiatric association Washington, DC 2013.

[24] Reas DL, Ro O. Less symptomatic, but equally impaired: Clinical impairment in restricting versus binge-eating/purging subtype of anorexia nervosa. Eat Behav. 2018;28:32–37. 10.1016/j.eatbeh.2017.12.004 29310054

[25] Ekeroth K, Clinton D, Norring C, Birgegård A. Clinical characteristics and distinctiveness of DSM-5 eating disorder diagnoses: findings from a large naturalistic clinical database. J Eat Disord. 2013;1:31. 10.1186/2050-2974-1-31 PMC4081791 24999410

[26] Forcano L, Alvarez E, Santamaría JJ, Jimenez-Murcia S, Granero R, Penelo E, et al. Suicide attempts in anorexia nervosa subtypes. Compr Psychiatry. 2011;52:352–8. 10.1016/j.comppsych.2010.09.003 21683172

[27] Calero-Elvira A, Krug I, Davis K, López C, Fernández-Aranda F, Treasure J. Meta-analysis on drugs in people with eating disorders. Eur Eat Disord Rev. 2009;17:243–59. 10.1002/erv.936 19475697

[28] Garner DM, Garner MV, Rosen LW. Anorexia nervosa “restricters” who purge: implications for subtyping anorexia nervosa. Int J Eat Disord. 1993;13:171–85. 10.1002/1098-108x(199303)13:2<171::aid-eat2260130205>3.0.co;2-l 8477286

[29] Deter H-C, Herzog W. Anorexia nervosa in a long-term perspective: results of the Heidelberg-Mannheim Study. Psychosom Med. 1994;56:20–27. 10.1097/00006842-199401000-00003 8197311

[30] Strober M, Freeman R, Morrell W. The long‐term course of severe anorexia nervosa in adolescents: Survival analysis of recovery, relapse, and outcome predictors over 10–15 years in a prospective study. Int J Eat Disord. 1997;22:339–60. 10.1002/(sici)1098-108x(199712)22:4<339::aid-eat1>3.0.co;2-n 9356884

[31] Van Autreve S, De Baene W, Baeken C, van Heeringen K, Vancayseele N, Vervaet M. Differential neural correlates of set-shifting in the bingeing-purging and restrictive subtypes of anorexia nervosa: An fMRI study. Eur Eat Disord Rev. 2016;24:277–85. 10.1002/erv.2437 26856396

[32] Murao E, Sugihara G, Isobe M, Noda T, Kawabata M, Matsukawa N, et al. Differences in neural responses to reward and punishment processing between anorexia nervosa subtypes: An fMRI study. Psychiatry Clin Neurosci. 2017;71:647–58. 10.1111/pcn.12537 28459134

[33] Miyake Y, Okamoto Y, Onoda K, Kurosaki M, Shirao N, Okamoto Y, et al. Brain activation during the perception of distorted body images in eating disorders. Psychiatry Res. 2010;181:183–92. 10.1016/j.pscychresns.2009.09.001 20153150

[34] Eddy KT, Dorer DJ, Franko DL, Tahilani K, Thompson-Brenner H, Herzog DB. Diagnostic crossover in anorexia nervosa and bulimia nervosa: implications for DSM-V. Am J Psychiatry. 2008;165:245–50. 10.1176/appi.ajp.2007.07060951 PMC3684068 18198267

[35] Serra R, Di Nicolantonio C, Di Febo R, De Crescenzo F, Vanderlinden J, Vrieze E, et al. The transition from restrictive anorexia nervosa to binging and purging: a systematic review and meta-analysis. Eat Weight Disord. 2022;27:857–65. 10.1007/s40519-021-01226-0 PMC8964622 34091875

[36] Bang L, Ro O, Endestad T. Normal gray matter volumes in women recovered from anorexia nervosa: a voxel-based morphometry study. BMC Psychiatry. 2016;16:144. 10.1186/s12888-016-0856-z PMC4866026 27177782

[37] Yue L, Wang Y, Kaye WH, Kang Q, Huang JB, Cheung EFC, et al. Structural alterations in the caudate nucleus and precuneus in un-medicated anorexia nervosa patients. Psychiatry Res Neuroimaging. 2018;281:12–18. 10.1016/j.pscychresns.2018.08.009 30212787

[38] Lazaro L, Andres S, Calvo A, Cullell C, Moreno E, Plana MT, et al. Normal gray and white matter volume after weight restoration in adolescents with anorexia nervosa. Int J Eat Disord. 2013;46:841–8. 10.1002/eat.22161 23904101

[39] Yu X, Robinson L, Bobou M, Zhang Z, Banaschewski T, Barker GJ, et al. Multimodal investigations of structural and functional brain alterations in anorexia and bulimia nervosa and their relationships to psychopathology. Biol Psychiatry. 2024;98:237–48. 10.1016/j.biopsych.2024.11.008 PMC7619009 39581292

[40] Wittchen HU. Strukturiertes klinisches Interview für DSM-IV : SKID. Achse I und II : Handanweisung. Hogrefe, Verlag für Psychologie; 1997.

[41] Panero M, Marzola E, Tamarin T, Brustolin A, Abbate-Daga G. Comparison between inpatients with anorexia nervosa with and without major depressive disorder: Clinical characteristics and outcome. Psychiatry Res. 2021;297:113734. 10.1016/j.psychres.2021.113734 33486276

[42] Hilbert A, Tuschen-Caffier B, Karwautz A, Niederhofer H, Munsch S. Eating disorder examination-questionnaire. Diagnostica. 2007;53:144–54.

[43] Ashburner J. A fast diffeomorphic image registration algorithm. Neuroimage. 2007;38:95–113. 10.1016/j.neuroimage.2007.07.007 17761438

[44] Ashburner J, Friston KJ. Unified segmentation. Neuroimage. 2005;26:839–51. 10.1016/j.neuroimage.2005.02.018 15955494

[45] Ashburner J, Friston KJ. Voxel-based morphometry–the methods. Neuroimage. 2000;11:805–21. 10.1006/nimg.2000.0582 10860804

[46] Smith SM, Nichols TE. Threshold-free cluster enhancement: addressing problems of smoothing, threshold dependence and localisation in cluster inference. Neuroimage. 2009;44:83–98. 10.1016/j.neuroimage.2008.03.061 18501637

[47] Zhang S, Wang W, Su X, Kemp GJ, Yang X, Su J, et al. Psychoradiological investigations of gray matter alterations in patients with anorexia nervosa. Transl Psychiatry. 2018;8:277. 10.1038/s41398-018-0323-3 PMC6293321 30546047

[48] McFadden KL, Tregellas JR, Shott ME, Frank GK. Reduced salience and default mode network activity in women with anorexia nervosa. J Psychiatry Neurosci. 2014;39:178–88. 10.1503/jpn.130046 PMC3997603 24280181

[49] Joos AA, Saum B, van Elst LT, Perlov E, Glauche V, Hartmann A, et al. Amygdala hyperreactivity in restrictive anorexia nervosa. Psychiatry Res: Neuroimaging. 2011;191:189–95. 10.1016/j.pscychresns.2010.11.008 21316204

[50] Müller VI, Langner R, Cieslik EC, Rottschy C, Eickhoff SB. Interindividual differences in cognitive flexibility: influence of gray matter volume, functional connectivity and trait impulsivity. Brain Struct Funct. 2015;220:2401–14. 10.1007/s00429-014-0797-6 PMC4981636 24878823

[51] Friederich HC, Herzog W. Cognitive-behavioral flexibility in anorexia nervosa. Curr Top Behav Neurosci. 2011;6:111–23. 10.1007/7854_2010_83 21243473

[52] Gaudio S, Wiemerslage L, Brooks SJ, Schiöth HB. A systematic review of resting-state functional-MRI studies in anorexia nervosa: Evidence for functional connectivity impairment in cognitive control and visuospatial and body-signal integration. Neurosci Biobehav Rev. 2016;71:578–89. 10.1016/j.neubiorev.2016.09.032 27725172

[53] Frank GK, Shott ME, Hagman JO, Yang TT. Localized brain volume and white matter integrity alterations in adolescent anorexia nervosa. J Am Acad Child Adolesc Psychiatry. 2013;52:1066–1075.e1065. 10.1016/j.jaac.2013.07.007 PMC4082770 24074473

[54] Weiner KS, Zilles K. The anatomical and functional specialization of the fusiform gyrus. Neuropsychologia. 2016;83:48–62. 10.1016/j.neuropsychologia.2015.06.033 PMC4714959 26119921

[55] Aminoff EM, Kveraga K, Bar M. The role of the parahippocampal cortex in cognition. Trends Cogn Sci. 2013;17:379–90. 10.1016/j.tics.2013.06.009 PMC3786097 23850264

[56] Vocks S, Busch M, Grönemeyer D, Schulte D, Herpertz S, Suchan B. Neural correlates of viewing photographs of one’s own body and another woman’s body in anorexia and bulimia nervosa: an fMRI study. J Psychiatry Neurosci. 2010;35:163–76. 10.1503/jpn.090048 PMC2861133 20420767

[57] Boehm I, King JA, Bernardoni F, Geisler D, Seidel M, Ritschel F, et al. Subliminal and supraliminal processing of reward-related stimuli in anorexia nervosa. Psychol Med. 2018;48:790–800. 10.1017/S0033291717002161 28832300

[58] Stopyra MA, Friederich HC, Mönning E, Lavandier N, Bendszus M, Herzog W, et al. The influence of homeostatic mechanisms on neural regulation of food craving in anorexia nervosa. Psychol Med. 2021;51:1011–9. 10.1017/S0033291719003970 PMC8161429 31931900

[59] Haber SN Frontiers in Neuroscience Neuroanatomy of Reward: A View from the Ventral Striatum. In: Gottfried JA (ed). *Neurobiology of Sensation and Reward*. Boca Raton (FL): CRC Press/Taylor & Francis Copyright © 2011 by Taylor and Francis Group, LLC., 2011. 22593898

[60] Rudebeck PH, Rich EL. Orbitofrontal cortex. Curr Biol. 2018;28:R1083–r1088. 10.1016/j.cub.2018.07.018 PMC9253859 30253144

[61] Frank GK. Altered brain reward circuits in eating disorders: chicken or egg? Curr Psychiatry Rep. 2013;15:396. 10.1007/s11920-013-0396-x PMC3888645 23963630

[62] Favaro A, Tenconi E, Degortes D, Manara R, Santonastaso P. Effects of obstetric complications on volume and functional connectivity of striatum in anorexia nervosa patients. Int J Eat Disord. 2014;47:686–95. 10.1002/eat.22320 24938422

[63] Cha J, Ide JS, Bowman FD, Simpson HB, Posner J, Steinglass JE. Abnormal reward circuitry in anorexia nervosa: A longitudinal, multimodal MRI study. Hum Brain Mapp. 2016;37:3835–46. 10.1002/hbm.23279 PMC5448464 27273474

[64] Frank GK, Shott ME, Riederer J, Pryor TL. Altered structural and effective connectivity in anorexia and bulimia nervosa in circuits that regulate energy and reward homeostasis. Transl Psychiatry. 2016;6:e932. 10.1038/tp.2016.199 PMC5314116 27801897

[65] Liu W, Zhan S, Li D, Lin Z, Zhang C, Wang T, et al. Deep brain stimulation of the nucleus accumbens for treatment-refractory anorexia nervosa: A long-term follow-up study. Brain Stimul. 2020;13:643–9. 10.1016/j.brs.2020.02.004 32289691

[66] Marsh R, Stefan M, Bansal R, Hao X, Walsh BT, Peterson BS. Anatomical characteristics of the cerebral surface in bulimia nervosa. Biol Psychiatry. 2015;77:616–23. 10.1016/j.biopsych.2013.07.017 PMC3933456 23978404

[67] Seitz J, Hueck M, Dahmen B, Schulte-Rüther M, Legenbauer T, Herpertz-Dahlmann B, et al. Attention network dysfunction in bulimia nervosa - An fMRI study. PLoS ONE. 2016;11:e0161329. 10.1371/journal.pone.0161329 PMC5015972 27607439

[68] Rosval L, Steiger H, Bruce K, Israël M, Richardson J, Aubut M. Impulsivity in women with eating disorders: problem of response inhibition, planning, or attention? Int J Eat Disord. 2006;39:590–3. 10.1002/eat.20296 16826575

[69] Siciliano L, Olivito G, Leggio M. The cerebellum gains weight: A systematic review of alterations in cerebellar volume and cerebro-cerebellar functional alterations in individuals with eating disorders. Neurosci Biobehav Rev. 2022;141:104863. 10.1016/j.neubiorev.2022.104863 36089105

[70] Zhu J-N, Wang J-J. The cerebellum in feeding control: Possible function and mechanism. Cell Mol Neurobiol. 2008;28:469–78. 10.1007/s10571-007-9236-z PMC11515829 18027085

[71] Brooks SJ, Barker GJ, O’Daly OG, Brammer M, Williams SC, Benedict C, et al. Restraint of appetite and reduced regional brain volumes in anorexia nervosa: a voxel-based morphometric study. BMC Psychiatry. 2011;11:179. 10.1186/1471-244X-11-179 PMC3278387 22093442

[72] Olatunji BO, Broman-Fulks JJ, Ciesielski BG, Zawilinski LL, Shewmaker S, Wall D. A taxometric investigation of the latent structure of eating disorders. Psychiatry Res. 2012;197:97–102. 10.1016/j.psychres.2011.12.016 22406392

[73] Murphy R, Straebler S, Cooper Z, Fairburn CG. Cognitive behavioral therapy for eating disorders. Psychiatr Clin North Am. 2010;33:611–27. 10.1016/j.psc.2010.04.004 PMC2928448 20599136

[74] Alzaid H, Simon JJ, Brugnara G, Vollmuth P, Bendszus M, Friederich H-C. Hypothalamic subregion alterations in anorexia nervosa and obesity: Association with appetite-regulating hormone levels. Int J Eat Disord. 2024;57:581–92. 10.1002/eat.24137 38243035

[75] Simon JJ, Stopyra MA, Mönning E, Sailer S, Lavandier N, Kihm LP, et al. Neuroimaging of hypothalamic mechanisms related to glucose metabolism in anorexia nervosa and obesity. J Clin Invest. 2020;130:4094–103. 10.1172/JCI136782 PMC7410066 32315289

[76] Frank GKW, Shott ME, Stoddard J, Swindle S, Pryor TL. Association of brain reward response with body mass index and ventral striatal-hypothalamic circuitry among young women with eating disorders. JAMA Psychiatry. 2021;78:1123–33. 10.1001/jamapsychiatry.2021.1580 PMC8246338 34190963

[77] Carr KD. Chronic food restriction: enhancing effects on drug reward and striatal cell signaling. Physiol Behav. 2007;91:459–72. 10.1016/j.physbeh.2006.09.021 17081571

[78] Oberndorfer TA, Frank GK, Simmons AN, Wagner A, McCurdy D, Fudge JL, et al. Altered insula response to sweet taste processing after recovery from anorexia and bulimia nervosa. Am J Psychiatry. 2013;170:1143–51. 10.1176/appi.ajp.2013.11111745 PMC3971875 23732817

[79] Kerr KL, Moseman SE, Avery JA, Bodurka J, Zucker NL, Simmons WK. Altered insula activity during visceral interoception in weight-restored patients with anorexia nervosa. Neuropsychopharmacology. 2016;41:521–8. 10.1038/npp.2015.174 PMC5130127 26084229

[80] Liu J, Xu X, Luo Q, Luo Y, Chen Y, Lui S, et al. Brain grey matter volume alterations associated with antidepressant response in major depressive disorder. Sci Rep. 2017;7:10464. 10.1038/s41598-017-10676-5 PMC5585337 28874763

