# Supplementary material for: Regional gray matter alterations in anorexia nervosa: implications for subtype heterogeneity and clinical severity
Source: Mol Psychiatry. 2026 Jul 15;31(12):6999–7009. doi: 10.1038/s41380-026-03757-3 (PMC13736514; doi:10.1038/s41380-026-03757-3)
Supplement: Supplementary file 1 [file 41380_2026_3757_MOESM1_ESM.docx]

**Supplementary material for:**

**Regional Gray Matter Alterations in Anorexia Nervosa: Implications for Subtype Heterogeneity and Clinical Severity**

Haidar Alzaid^1*^, Martin Bendszus^1^, Hans-Christoph Friederich^2^, Joe J. Simon^2^.

*Corresponding Author:

^1^Department of Neuroradiology, Heidelberg University Hospital, Heidelberg, Germany.

^2^Department of General Internal Medicine and Psychosomatics, University Hospital Heidelberg, Heidelberg, Germany.

Haidar Alzaid

Department of Neuroradiology

Im Neuenheimer Feld 400

69120 Heidelberg

Germany

**Supplementary Table S1. Antidepressant intake in the AN group:**

| **n (%) taking** **antidepressants** | **Antidepressant class** | **Agent (n)** | **Mean daily dose (mg ± SD)** | **Dose range (mg)** |
| --- | --- | --- | --- | --- |
| 10 (12.8%) | SSRI | Citalopram (1) | 30 ± 0 | 30 |
|  |  | Escitalopram (2) | 10 ± 0 | 10 |
|  |  | Fluxetine (1) | 20 ± 0 | 20 |
|  |  | Sertraline (2) | 100 ± 70.7 | 50 - 150 |
|  | SNRI | Ventlafaxine (1) | 75 ± 0 | 75 |
|  | NaSSA | Mirtazapine (4) | 18.8 ± 7.5 | 15 – 30 |

SSRI = selective serotonin reuptake inhibitor; SNRI = serotonin–norepinephrine reuptake inhibitor; NaSSA = noradrenergic and specific serotonergic antidepressant.

Values reflect medication status at the time of MRI scanning. *One patient was receiving a combination of venlafaxine and mirtazapine.*

**Supplementary Table S2. Psychiatric comorbidities in the entire AN group and by subtype (restricting vs. binge–purging):**

| **Parameter** | **AN (*n* = 78)** | **AN-R (*n* = 57)** | **AN-BP (*n* = 21)** | **AN-R vs. AN-BP (*p* value)** |
| --- | --- | --- | --- | --- |
| Comorbidities (total) | 19 (24.4%) | 12 (21.1%) | 7 (33.3%) | ns |
| Depression | 19 (24.4%) | 12 (21.1%) | 7 (33.3%) | ns |
| Panic disorder | 2 (2.6%) | 2 (3.5%) | 0 (0%) | ns |
| Obsessive–compulsive disorder | 1 (1.3%) | 1 (1.8%) | 0 (0%) | ns |

Values are given as n (%). Group differences between AN-R and AN-BP were assessed using chi-square tests. Abbreviations: AN, anorexia nervosa; AN-R, anorexia nervosa – restrictive subtype; AN-BP, anorexia nervosa - binge/purge subtype; ns, not significant. *Two subjects have more than one comorbidity.*

**Supplementary Table S3. Distribution of acquisition protocols across groups:**

| **Group** | **n** | **Protocol 1 (TR = 1570 ms, TE = 2.63 ms)** | **Protocol 2 (TR = 1900 ms, TE = 2.52 ms)** |
| --- | --- | --- | --- |
| **HC** | 94 | 69 (73.4 %) | 25 (26.6 %) |
| **AN (total)** | 78 | 54 (69.2 %) | 24 (30.8 %) |
| **AN-R** | 57 | 34 (59.6 %) | 23 (40.4 %) |
| **AN-BP** | 21 | 20 (95.2 %) | 1 (4.8 %) |

The distribution of acquisition protocols did not differ between AN and HC (χ² test, p > .05).

A significant difference was observed between AN-R and AN-BP (χ² test, *p* < .01).

**Supplementary Figure S1 and Table S4. Negative correlation between regional GMV and BMI in AN-BP:**

| **Peak region** | **MNI coordinates** | ***p* value (FWE-corrected)** | **Cluster size**  **(voxels)** | **Cluster regions** |
| --- | --- | --- | --- | --- |
| Olfactory cortex (R) | 6 x 6 x -18 | .021 | 202 | Olfactory cortex (R); nucleus accumbens (R); gyrus rectus (R); medial orbital gyrus (R); posterior orbital gyrus (R). |

Each significant cluster is defined by the region with the peak *p* value, and all regions within the clusters are listed according to the AAL3 atlas and verified through visual inspection. Abbreviations: AN-BP, anorexia nervosa - binge/purge subtype; BMI, body mass index; R, right hemisphere; L, left hemisphere.
